# Investigating Activity Recognition for Hemiparetic Stroke Patients Using Wearable Sensors: A Deep Learning Approach with Data Augmentation

**DOI:** 10.3390/s24010210

**Published:** 2023-12-29

**Authors:** Youngmin Oh, Sol-A Choi, Yumi Shin, Yeonwoo Jeong, Jongkuk Lim, Sujin Kim

**Affiliations:** 1 School of Computing, Gachon University, Seongnam 13120, Republic of Korea; youngminoh@gachon.ac.kr; 2 Department of Physical Therapy, Jeonju University, Jeonju 55069, Republic of Korea; sola1939@jj.ac.kr (S.-A.C.); sum4287@jj.ac.kr (Y.S.); zmxn971211@jj.ac.kr (Y.J.); 3 Department of Computer Engineering, Dankook University, Yongin 16890, Republic of Korea; lim.jeikei@dankook.ac.kr

**Keywords:** activities of daily living, classification, hemiparesis, human action recognition, range of motion, stroke rehabilitation, upper extremity, deep learning

## Abstract

Measuring the daily use of an affected limb after hospital discharge is crucial for hemiparetic stroke rehabilitation. Classifying movements using non-intrusive wearable sensors provides context for arm use and is essential for the development of a home rehabilitation system. However, the movement classification of stroke patients poses unique challenges, including variability and sparsity. To address these challenges, we collected movement data from 15 hemiparetic stroke patients (Stroke group) and 29 non-disabled individuals (ND group). The participants performed two different tasks, the range of motion (14 movements) task and the activities of daily living (56 movements) task, wearing five inertial measurement units in a home setting. We trained a 1D convolutional neural network and evaluated its performance for different training groups: ND-only, Stroke-only, and ND and Stroke jointly. We further compared the model performance with data augmentation from axis rotation and investigated how the performance varied based on the asymmetry of movements. The joint training of ND + Stroke yielded an increased F1-score by a margin of 31.6% and 10.6% compared to ND-only training and Stroke-only training, respectively. Data augmentation further enhanced F1-scores across all conditions by an average of 11.3%. Finally, asymmetric movements decreased the F1-score by 25.9% compared to symmetric movements in the Stroke group, indicating the importance of asymmetry in movement classification.

## 1. Introduction

### 1.1. Motivation

Hemiparesis, marked by the impairment of one side of the body due to a stroke, often results in reduced use of the more affected limb [1,2]. A substantial majority of stroke survivors (>85%) experience incomplete upper extremity (UE) motor recovery [3]. This often results in sustained low engagement of the more affected limb in activities of daily living (ADL) in the home setting following hospital discharge [4,5]. Encouraging the repetitive use of the more affected upper limb for ADL is a crucial aspect of rehabilitative training, not only facilitating functional improvement but also initiating a virtuous cycle of increased spontaneous use [6]. In this context, the combination of wearable sensors and advanced deep learning methodologies presents a new approach for monitoring UE usage, providing invaluable feedback on limb use [7,8].

There exist various methods for the monitoring of patient activities, including inertial measurement units (IMUs), accelerometers, home video surveillance, and utilization of applications for patient responses at designated times [9,10,11]. Accelerometers allow the relative assessment of the movement of the more affected upper limb compared to the less affected limb; however, the raw data do not directly provide insights into the specific movements performed [12]. Video surveillance has potential issues such as privacy infringement; moreover, it restricts the patient’s location within the camera’s recording field if the camera position is fixed [13]. Extended monitoring of patients’ activities via applications is challenging and usually depends on the patient’s willingness to participate [11]. Furthermore, this makes it difficult to precisely identify the type of upper limb movement performed by the patient. IMUs have limitations similar to accelerometers; however, recent advancements in human activity recognition technology have enabled the classification of specific upper limb movements using the raw time series data of IMU sensor channels [14]. The wide availability of embedded IMU sensors in commercial wearables, such as smartwatches, makes an IMU sensor-based algorithm or model easily deployable without relying on a special device designed for a specific purpose.

### 1.2. Related Work

Traditionally, time series data from wearable sensors are preprocessed and feature-engineered to be fed into machine learning algorithms for activity classification [15]. Feature engineering is typically handcrafted based on the researchers’ domain expertise, and it can be categorized into time-domain features [7,8,16], such as mean, variance, correlation, min, max, and skewness, and frequency-domain features after the original time series is transformed into a frequency domain [17,18]. However, feature engineering can be subjective and may not generalize properly to unseen data distributions or novel movement classes. Therefore, researchers are adopting deep learning methods to classify the movements of stroke patients and non-disabled individuals [19,20,21]. In a deep learning architecture comprising multiple layers, the initial layers work as a series of hierarchical feature extractors; however, the actual features or model parameters are solely learned from the data, which boosts the model performance and generalizability. For human activity recognition (HAR), one-dimensional convolutional neural networks (1D-CNN) [21,22] and recurrent neural networks [23,24] are the most common architectures used to extract temporal patterns from sensor time series.

Compared with the HAR of non-disabled individuals, recognizing the movements of patients with stroke presents unique challenges. First, the movements of stroke patients are distinctive and diverse compared to those of non-disabled individuals, thus making it ineffective to train a model with non-disabled participants’ data and deploy it to classify the movements of patients with stroke. However, training a model using only stroke patients’ data is challenging because of data sparsity [25]. Nonetheless, researchers have mostly used only the data of patients with stroke, which are relatively small in terms of the number of participants and movement types. For example, O’Brien et al. [7] investigated gait-related activity recognition in patients with gait-impaired stroke and in healthy participants using smartphone sensors. They found that when evaluating performance in the stroke group, their random forest classifier performed better when trained with stroke patients’ data than with healthy participants’ data. Second, stroke patients’ movements are heterogeneous not only in their patterns but also in their duration. The same movement can have different durations depending on the individual’s status, and different movements require different completion times. This variability in duration is a significant challenge, especially for a deep learning model, because the input signal usually requires a fixed length or dimension. Third, the upper limb movement patterns of stroke patients are differently affected in terms of the bilaterality and asymmetry of a given movement. Therefore, distinguishing a movement’s bilaterality and asymmetry is important not only for activity classification but also for effective rehabilitation [26,27,28]. However, most previous studies on activity recognition did not consider how movement asymmetry affects classification performance. Bailey et al. [29] measured bilateral upper limb activity and the ratio of non-disabled adults to stroke patients using wrist-worn accelerometers. However, their measurement of bilateral asymmetry was continuously recorded for approximately a day and did not differentiate which activity was performed.

## 2. Materials and Methods

In the present study, to investigate an efficient method for recognizing stroke patients’ movements in daily life, we collected movement data from hemiparetic stroke patients and non-disabled individuals. The participants performed two distinct tasks: a range of motion (ROM) task that involves isolated unimanual movements and an ADL task comprising daily activities in a home-like environment. To address the above-mentioned challenges of variability and asymmetry, we first fixed the length of the input signal using linear interpolation. Afterward, we trained a deep learning model using different combinations of training groups (ND and Stroke) and compared the results. Furthermore, we adopted a data augmentation technique for time series data [30] to further enhance the performance. Finally, we investigated how the asymmetry of movements affects model performance when evaluated using stroke data. These methods and findings provide practical guidelines for classifying the movements of stroke patients when using deep learning models, which is essential in developing a home rehabilitation system. Furthermore, to facilitate further research, we open-sourced our dataset, Jeonju University IMU human action recognition (JU-IMU; https://github.com/youngminoh7/JU-IMU, accessed on 23 November 2023).

### 2.1. Experiments

#### 2.1.1. Participants

Fifteen patients with subacute to chronic stroke (four females; Stroke group) and 29 non-disabled (ND) young volunteers (19 females; ND group) participated in this study (Table 1). Nine patients had right hemiparesis and six patients had left hemiparesis. The inclusion criteria for the Stroke group were as follows: (1) age ≥ 21, (2) ischemic or hemorrhagic stroke in the subacute to chronic stage, (3) impaired upper extremity movement (Fugl–Meyer Assessment for Upper Extremity (FMA-UE) score ≥ 19 out of 66), (4) intact cognitive functions to understand the experimental process and communicate with the instructor (Korean version of Mini-Mental State Exam score > 24), and (5) absence of vision problems or previous orthopedic surgeries on the upper extremity that would interfere with movement [31]. The inclusion criteria for the ND group were (1) age ≥ 21 and (2) no history of neurological or orthopedic surgeries that would affect the movement of the upper extremities. All participants were right-handed (or right-handed before the stroke in the Stroke group), as assessed using the Edinburgh Handedness Inventory [32]. The Institutional Review Board of Jeonju University approved this study (jjIRB-171115-HR-2017-1109).

#### 2.1.2. Selection of Upper Extremity Movements

The movements selected for the experiment included movements of the upper extremities during ADL in various locations at home (e.g., bathroom, bedroom, living room, or kitchen), as well as motions used for clinical tests, such as the FMA-UE, the Wolf Motor Function Test (WMFT), and the Actual Amount of Use Test (AAUT) [33,34,35]. These selected movements were divided into two tasks: the ROM and ADL tasks. The ROM task comprised simple ROM movements at each joint of the upper extremity, such as the shoulder (flexion/extension, abduction/adduction, and internal rotation), elbow, and wrist (flexion/extension), and grasping of small items. Meanwhile, the ADL task included movements in ADL such as combing hair, opening a book, and folding a towel [2,34,36]. These activities require purposeful movements at different locations in the home. Movements in the ADL task were further divided into three movement types: unimanual (UNI), bimanual asymmetric (BIA), and bimanual symmetric (BIS). UNI movements involved the use of one arm by the participants, whereas BIA and BIS movements involved the use of both arms simultaneously but with different symmetries. In BIA movements, each arm and hand played a specific role in movement control (e.g., holding a paper with the left hand and writing with the right), whereas in BIS movements, both arms moved symmetrically. A panel of two physical therapists and one occupational therapist classified UNI, BIA, and BIS movements based on a previous study [1]. When stroke patients performed BIA movements, we instructed them to use the more affected limb for major or delicate movements and the less affected limb for the holding of objects. For example, in the movement of writing on paper, patients with right hemiparesis used their right hand to write with a pen and their left hand to hold the paper; patients with left hemiparesis used the opposite limb, although all were right-handed before the onset of stroke.

Table 2 summarizes the information regarding our movement sets, and Tables S1 and S2 in the Supplementary Materials describe all movements in the ROM and ADL tasks. Most selected movements were from clinical tests based on the International Classification of Function and Disability (ICF Model). To assess the level of impairment, items in the FMA-UE were used; to assess the activity level, WMFT, action research arm test, arm motor ability test, motor evaluation scale for the upper extremities in stroke patients, and “Test D’evaluation des Membres Suprrieurs des Personnes Agées” were used. For the participation level, the French Activities Index, Barthel index, Functional independence measure, Motor Activity Log, and AAUT were used [37].

#### 2.1.3. Experimental Environments

**Figure 1:**
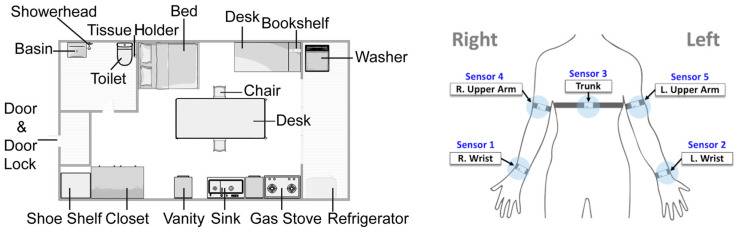
Experimental studio space (**left panel**) and sensor attachment locations (**right panel**). The studio was divided into sleeping areas, study room, bathroom, veranda, kitchen, and living room, distinguished by the arrangement of furniture. Participants wore five sensors on their bodies (two on the wrists, two on the upper arms, and one on the trunk) and performed range of motion and activities of daily living tasks according to a predetermined scenario. The experimenters demonstrated each movement to the participants in advance, and the participants were instructed to replicate that movement exactly.

To encourage the participants to perform realistic movements at home, we set up a dedicated studio for the experiment (Figure 1, left panel). This studio had a single room that included a restroom, veranda, kitchen, and an entrance hall with a shoebox. We ensured the availability of the necessary materials for each movement, such as a set of chopsticks for handling chopsticks and dishes for washing. Additionally, we provided the appropriate furniture, including desks, chairs with backrests, refrigerators, closets, sinks, laundry machines, clothes dryers, and vacuum cleaners, as required for each movement. These provisions allowed the participants to perform actions in accordance with the scenario provided.

#### 2.1.4. Sensors

We used five IMU sensors to collect data (Figure 1, right panel). These sensors were 50 × 100 × 20 mm^3^ and weighed 200 g. The IMU sensors included an accelerometer, a gyroscope, and a magnetometer, each with three perpendicular axes (x, y, and z). Three YI 4 K Action Cameras (YI Technology, Shanghai, China) were mounted on the walls of the studio to record the experiment. Video recordings were combined with IMU sensors to segment and annotate the participants’ upper limb movements. During the experiment, all the participants wore IMU sensors at five specific body locations: the wrists and upper arms on both sides of the upper limb and the center of the trunk (Figure 1, right panel). To ensure consistency, each sensor was placed in the same location for all participants. Specifically, sensor 1 was positioned on the right wrist, sensor 2 on the left wrist, sensor 3 on the trunk, sensor 4 on the right upper arm, and sensor 5 on the left upper arm. The IMU sensors had a mean sampling rate of 80 Hz and transmitted the IMU signal wirelessly to a smartphone via Bluetooth. The data were saved as comma-separated value (CSV) files, where each row contained a timestamp and sensor values from 45 channels (five sensors × nine channels: x, y, z for the accelerometer, gyroscope, and magnetometer). Similarly, video data captured by the action cameras were saved on a mobile phone using Bluetooth wireless technology. Following the completion of the experiment, the CSV and video files were exported from the mobile phone to a computer for further analysis.

#### 2.1.5. Experimental Procedures

The participants were engaged in the experiment for a maximum of 1–2 h. The IMU sensors were attached to five body locations using elastic bands. Participants sequentially performed movements in the ROM and ADL tasks, repeating each movement five times. All the participants followed the same order of movement for both tasks. To clearly mark the start and end of each movement, the participants clapped their hands between movements. Hand clapping was also used to annotate each movement. The experiment was conducted by three researchers. One researcher verbally explained the motion, the second demonstrated the motion, and the third monitored the video and real-time data stream from the IMU sensors. ND participants were given a 5 min rest period between tasks, whereas stroke patients had a maximum rest period of 10 min. However, if the participants reported fatigue, they were allowed to take a break at any time or stop the experiment. In these cases, the experiment was resumed on another day to complete the task.

### 2.2. Data Analysis

#### 2.2.1. Data Annotation and Exclusion

A custom *Matlab* code (Mathworks, Natick, MA, USA) was developed to segment and annotate each movement based on the synchronized IMU and video data. The raw data from the IMU sensors (in CSV format) were synchronized with the video recordings that captured all the movements during the experiment. The researchers manually labeled each movement by observing the synchronized IMU and video data. Erroneous trials in which participants were unable to successfully complete the motions (e.g., scratching one’s nose or hair due to tickling or performing the wrong sequence) were excluded.

Additionally, because we trained and evaluated the model on an individual basis, we excluded a participant’s data if they missed more than one movement segment. Missing segments occurred during the experiment either because a participant could not perform a movement or because the recorded data were corrupted during the transmission process. Of the 15 patients in the Stroke group, data from one participant (Stroke11) were excluded on both ROM and ADL tasks. Of the 29 participants in the ND group, the data of two participants (ND9 and ND13) were excluded from the ROM task (N = 27), and the data of one participant (ND10) were excluded from the ADL task (N = 28).

#### 2.2.2. Preprocessing and Asymmetry Score

Each movement segment consisted of five repetitions. For each segment, we extracted the segment means from all channels and subtracted the channel means from the channel values, making the segment means of all the channels zero. For the subsequent analyses, we took six out of nine channels from each sensor—x-, y-, and z-axes of the accelerometer and gyroscope, excluding magnetometer channels—because these values depend on the orientation of the participants relative to the Earth’s magnetic fields.

To estimate the asymmetry of movements, we defined an asymmetry score that measures the log ratio of the right-side energy to the left-side energy. The right-side energy $E_{R}$ and left-side energy $E_{L}$ are defined as follows:

(1) $$E_{R}=\frac{1}{N_{R}}\underset{c\;\in \;R_{C}}{\sum }\overset{T}{\underset{t=1}{\sum }}\frac{1}{T}{\left(x_{c,\;t}\right)}^{2},\;\;E_{L}=\frac{1}{N_{L}}\underset{c\;\in \;L_{C}}{\sum }\overset{T}{\underset{t=1}{\sum }}\frac{1}{T}{\left(x_{c,\;t}\right)}^{2}\;,$$

where $N_{R}$ and $N_{L}$ are the number of channels *c* corresponding to the right-side $R_{C}$ and left-side $L_{C}$; $x_{c,\;t}$ is the zero-averaged value of channel c at time t; and T is the length (time points) of a segment, which is a constant across all segments. The asymmetry score $S_{asym}$ is defined as

(2) $$S_{asym}=log\left(\frac{E_{R}+\epsilon }{E_{L}+\epsilon }\right)\;,\;$$

where *log* is the natural logarithm and ϵ=0.001 is a small positive constant to make the computation stable when the denominator is close to 0. By definition, $S_{asym}$ is positive and increases when $E_{R}$ is larger than $E_{L}$, zero when $E_{R}=E_{L}$, and negative and decreases when $E_{R}$ is smaller than $E_{L}$. We will sometimes report the absolute value of $S_{asym}$ in the Results section when only the degree of asymmetricity is important, regardless of which side’s energy is larger. Patients in the ND group and patients with right hemiparesis used their dominant right hands when performing the UNI and BIA movements. In contrast, patients with left hemiparesis performed the same movements with their affected, non-dominant left hand and were expected to have negative asymmetry scores. We exchanged the sensor numbers between the right and left sides when the sensor data were fed into the neural network model for training and evaluation. We performed this process to make the data distribution among the sensors similar across all participants for easy capture of the dominant patterns by the model.

#### 2.2.3. Linear Interpolation and Sliding Windows

**Figure 2:**
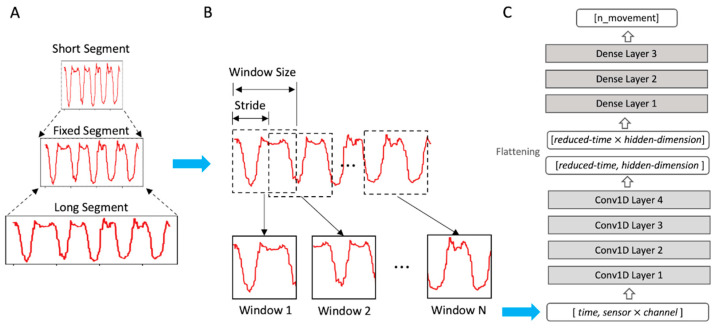
Data preprocessing and the model structure. For brevity, only a single channel time-series (the red curves) is shown as a demonstration. The horizontal blue arrows indicate the flow of data processing. (**A**) Linear interpolation. Segments of varying lengths are interpolated to have a fixed length. (**B**) Sliding windows. The fixed-length segment is sliced into multiple windows that move with a length of stride. (**C**) The model structure. The gray boxes represent layers of the neural network, and the white boxes represent the processed data dimensions.

The segments of different movements of different participants have various lengths in terms of time points. To assign a fixed-length input to a neural network model, we applied the sliding window technique that slices a segment into several pieces or “windows” (Figure 2). Starting from the beginning of a segment, a sliding window cuts out a fragment of the segment with a fixed length called “window size”, and then it jumps forward by a step length called a “stride” until the remaining length is less than the window size. However, there are two critical problems in applying the sliding window technique to our data with variable segment lengths. First, for a fixed window size, shorter segments produce fewer segments, whereas longer segments result in more segments. This leads to a class imbalance in machine learning, which causes biased learning to favor classes with larger sample sizes, making objective evaluation difficult. Second, a fixed window size would capture different proportions of the original segment. The same length window contains a relatively smaller portion of a longer segment and vice versa. This makes it difficult for a neural network model to discover common temporal patterns from variable-length segments of the same activity. These problems are particularly critical for our dataset because the inter-participant and inter-activity segment lengths vary significantly (see the Section 3.1). To solve the issue of variable segment lengths, we adopted a simple linear interpolation to ensure that all segments had the same average length. After linear interpolation, we applied a sliding window to obtain the same number of windows for all the segments. The window size and stride were determined to produce exactly 20 windows per segment for ROM and ADL tasks.

#### 2.2.4. Model

We adopted the 1D-CNN [22] to classify participants’ movement in our dataset. The 1D-CNN applies convolution along the temporal dimension to the time series of the sensor values. Specifically, we shaped the window data to have dimensions of [time, channel], where the channel corresponded to 30 channels from a combination of five sensors and six corresponding channels (three accelerometer channels and three gyroscope channels). As shown in Figure 2, we stacked four convolutional layers followed by three dense layers for movement classification. The four convolutional layers function as feature extractors, and each convolutional layer extracts local temporal patterns from the input features. For all the convolutional layers, the size of the convolution kernel was five, and the stride of the kernel was two. The numbers of kernels and output features of the convolution layers were 32, 64, 128, and 256 for the four layers, respectively. These progressively increasing numbers of features were inspired by 2D convolutional neural networks [39,40]. For the ROM task, with an input window length of 200, the lengths of the features became 98, 47, 22, and 9 after being transformed by each convolutional layer. The corresponding feature lengths for the ADL task with an input window length of 740 were 368, 182, 89, and 43. With this design of increasing feature numbers and shrinking feature lengths, convolutional layers can transform the input data into more diverse and abstract representations in the latter layers, which makes them efficient feature extractors. After the features were extracted by the convolutional layers, they were flattened and fed into the dense layers that work as classifiers. The first two dense layers contained 800 and 200 neurons, respectively. The final dense layer had a neuron number equal to the number of movements: 14 for the ROM task and 56 for the ADL task and was activated using the softmax function. All layers, including the convolution layers and the last dense layer, were activated using a rectified linear unit (ReLU) [41]. Additionally, the first two dense layers were applied with Dropout [42] with a probability of 0.7.

#### 2.2.5. Data Augmentation

**Figure 3:**
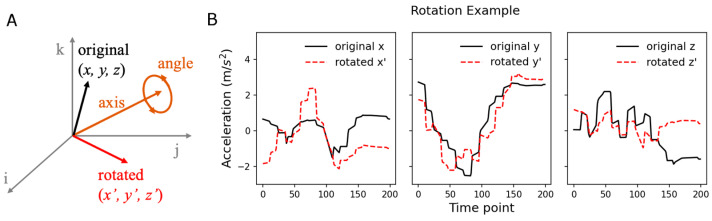
Axis rotation. (**A**) Visualization of axis rotation. The sensor values form a vector (“original”) and are rotated by a random angle with respect to a randomly chosen axis. (**B**) Rotation example. Black solid lines indicate x, y, and z components of an accelerometer and red dashed lines denote rotated components.

Data augmentation applies certain transformations to the original data to increase the amount of training data by supplementing uncovered space in the feature space. Data augmentation has been widely adopted in other fields of deep learning such as image recognition, comprising random translation, resizing, cropping, flipping, and rotation [43]. These geometrical transformations on image datasets improve model training, resulting in better generalizability to unseen images during testing. However, it is unclear what types of transformations are beneficial for human activity recognition using time series data, although some studies [30] have suggested certain categories of transformations, including scaling, rotation, permutation, and cropping, may work for time series-based action recognition. The extent of improvements associated with these transformations depends on the type of motion and structure of the dataset. Our dataset consisted of complex ADL activities, often exhibiting strong asymmetry and diversity among different participants. These characteristics encouraged us to apply axis rotation as a method of data augmentation. The axis rotation rotated sensor values by a random angle (sampled from −90 to +90 degrees) with respect to a randomly selected axis in a 3-dimensional space that the x, y, and z components of sensor values constitute (Figure 3). The axis rotation simulates the variability in hand and arm orientations during movements. To evaluate the performance boost of data augmentation, we trained our model using two different datasets: the original dataset and the original dataset plus its randomly rotated dataset (the augmented set).

#### 2.2.6. Training and Evaluation

**Figure 4:**
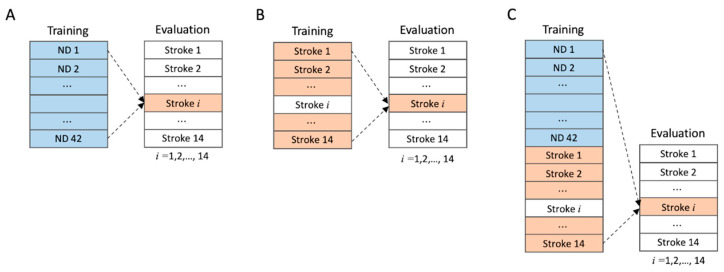
Training and evaluation conditions. Subject boxes filled with colors are used for training/evaluation, and those unfilled (white background) are not used. Evaluation metric is calculated for each subject in the Stroke group and then averaged for all subjects. (**A**) Training with ND (Split). (**B**) Training with Stroke (LOSO-CV). (**C**) Training with ND + Stroke (LOSO-CV).

As this study aimed to enhance the classification of the movements of stroke patients, we first examined how different combinations of ND and Stroke training groups affected the evaluation results of stroke patients. Therefore, according to the study design, the Stroke group must be included in both the training and evaluation sets. Because any machine learning model should not have the same data in the training and evaluation sets, we adopted leave-one-subject-out cross-validation (LOSO-CV) as the main evaluation method (Figure 4). In LOSO-CV, only one participant from the target group (the Stroke group in our case) is kept from the training group, and the model performance was evaluated using the remaining participant after the model was trained using the other participants’ data. This process was repeated as many times as the number of participants, and the evaluation result for each participant was averaged to obtain the LOSO-CV evaluation result. For our study design, we tested three different training conditions: ND only, Stroke only, and ND + Stroke. For ND-only training, the training group was the ND group and the evaluation group was the Stroke group, in which the ordinary separate training and test groups were applied without the use of LOSO-CV. For Stroke-only training, the training group comprised only the Stroke group, and LOSO-CV was applied. Finally, for the ND + Stroke training, the training group was a combination of the ND and Stroke groups, whereas the evaluation was estimated in the Stroke group using LOSO-CV.

For optimization, we adopted the AdamW optimizer (learning rate = 0.001, betas = (0.9, 0.999), eps = 1 × 10^−8^, and weight decay = 0.01) [44] with a cross-entropy loss. The batch size was 256 (windows) and the training epoch was 40. The training results were evaluated by F1-score on individual movement segments for each participant as the harmonic mean of precision and recall for each class. Thus, we have one F1-score corresponding to each pair of participants and classes. Group-averaged F1-scores were calculated based on patient groups and movement types.

#### 2.2.7. Statistical Analysis

To determine the impact of the training group and augmentation method on model performance, a mixed-effect model with categorical variables [45] was used in the R software package (version 4.3.2) [46]. For each ROM and ADL task, the training groups (ND and Stroke) and augmentation conditions (the original and the augmented data) were set as categorical variables of the fixed effects, and each participant was set as a random intercept. Four analytical models were constructed as follows: The first two models included only one factor (training group or augmentation), whereas the last two had two factors with and without interaction terms (e.g., training group + augmentation for Model 3, training group × augmentation for Model 4). To define the best-fit model, the ANOVA function in R was applied. When necessary, Tukey’s post hoc test was performed. In addition, the effect of movement types (UNI, BIA, and BIS) in the ADL task on F1-scores across the training groups was studied via linear regression, where the factors were movement types (BIA, UNI, and BIS) and training groups (ND and Stroke). All significance levels were set at *p* < 0.05.

## 3. Results

### 3.1. Data Exploration

**Figure 5:**
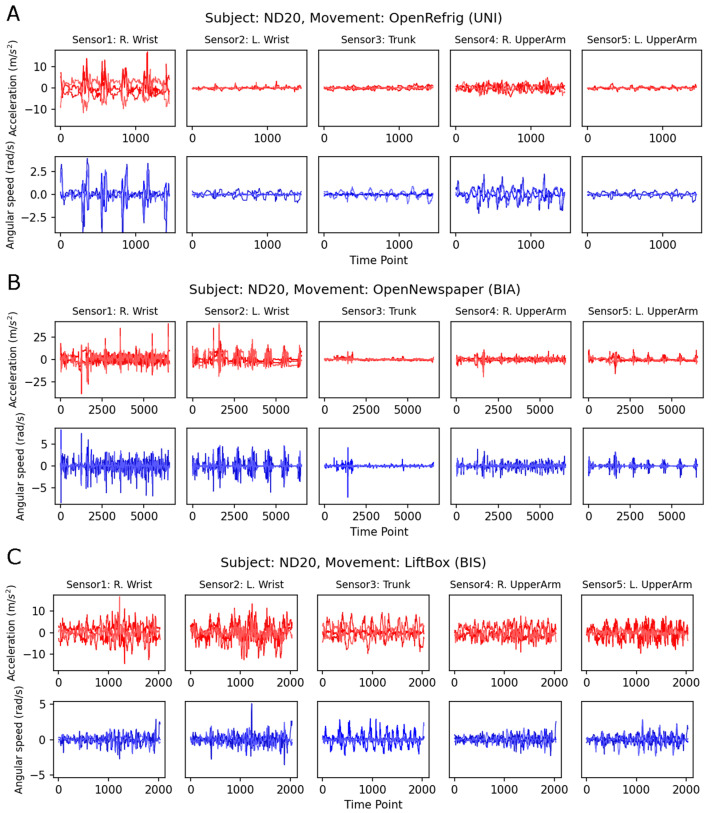
Sample plots of three movements of the same participant (ND20) in the ADL task. (**A**) OpenRefrig—UNI, (**B**) OpenNewspaper—BIA, (**C**) LiftUpDownBox—BIS. Red lines: accelerometer channels, blue lines: gyroscope channels. Note that the scales of the horizontal (time points) and vertical (sensor values) axes across panels are different. OpenRefrig: open and close refrigerator, OpenNewspaper: open a newspaper; LiftBox: lift the box up and down; UNI: unimanual; BIA: bimanual asymmetric, BIS: bimanual symmetric; R: right; L: left.

First, we present sample plots of the segments annotated from the ADL task (Figure 5). Sample plots in Figure 5 provide examples of three different types of movements—UNI, BIA, and BIS—by the same participant (ND20). The asymmetric nature of each movement type was reflected in the magnitudes and patterns on both sides of the sensors.

**Figure 6:**
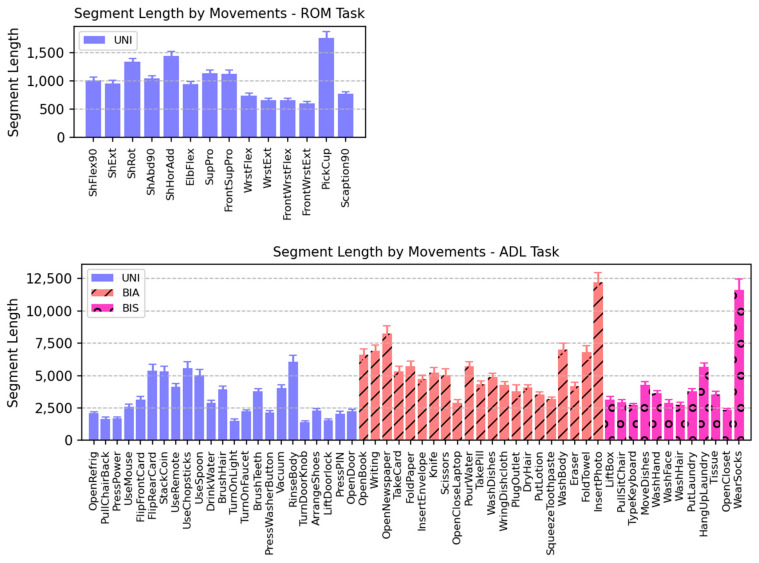
Average segment length of each movement of all participants. Segment length is in time points. Error bars represent standard errors. Each segment consists of five repetitions of the same movement. Movements are divided into three groups according to their asymmetricity: UNI, BIA, and BIS. UNI, unimanual; BIA, bimanual asymmetric; BIS, bimanual symmetric. Note that scales of the vertical axes are different between the two tasks. (**Up**) ROM task. (**Down**) ADL task.

Table 3 summarizes the segment length statistics. For the ROM task, 41 participants × 14 movements produced 574 segments. For the ADL task, 42 participants × 56 movements made 2352 segments, but there were six missing segments from the data. Therefore, the total number of segments was 2352 − 6 = 2346 segments. Figure 6 shows the variability in the segment lengths of movements. The ROM task had relatively uniform segment lengths compared with the ADL task because its movements were simpler and less variable. However, the movements in the ADL task varied significantly in terms of length and pattern. The maximum segment length of the ADL task was approximately 100 times the minimum length on an individual-segment basis. This significant variability in segment lengths was the main reason we applied linear interpolation to adjust the segment lengths to a fixed value close to the mean length. The adjusted segment length was fixed at 1000 time points for the ROM task and at 3700 time points for the ADL task. The interpolated segment length was intended to produce exactly 20 windows per segment. The corresponding window size and stride length were 200 and 42 for the ROM task and 740 and 150 for the ADL task, respectively.

**Figure 7:**
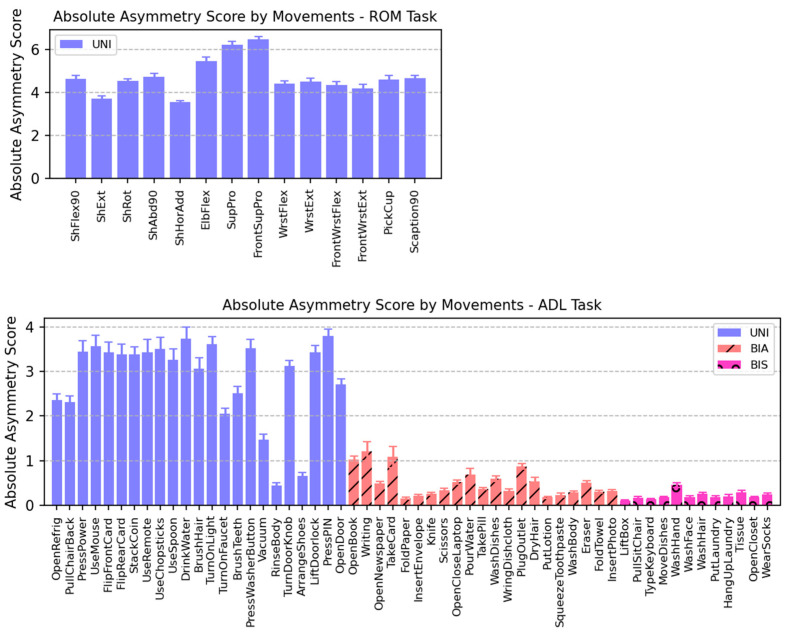
Absolute asymmetry score (AAS) of each movement averaged across participants. Regardless of right- or left-dominated, higher AAS indicates more asymmetrical movements, whereas lower AAS represents symmetrical movements in terms of energy. Note that scales of the vertical axes are different between the two tasks. (**Up**) ROM task. (**Down**) ADL task. UNI, unimanual; BIA, bimanual asymmetric; BIS, bimanual symmetric.

Table 4 summarizes the asymmetry of movements represented by the absolute asymmetry score (AAS), and Figure 7 shows the mean AAS across participants. As expected, more asymmetrical movements resulted in higher AAS. Among the three movement types, the UNI movement had the highest AAS, followed by the BIA and BIS movements. It is important to note that “symmetry” is primarily determined by the relative energy level of both sides (Equations (1) and (2)) and may not necessarily reflect similarities in movement patterns. Therefore, some movements had distinct AAS values compared with other movements in their group.

### 3.2. Training Results

Table 5 summarizes the mean F1-scores evaluated in the Stroke group using three different combinations of training groups: ND, Stroke, and ND + Stroke training groups. F1-scores were averaged across all movements for each participant in either task (ROM or ADL). In the ROM task, ND + Stroke or Stroke training performed better than ND training (*p* < 0.001 for both groups), but there was no significant difference between the ND + Stroke and Stroke groups (*p* = 0.164). In contrast, performance improved in the following order: ND, Stroke, and ND + Stroke in the ADL task. Specifically, ND + Stroke training performed significantly better than Stroke (*p* = 0.003) or ND (*p* < 0.001). Stroke training also showed a better performance than ND training (*p* < 0.001). In addition, the augmented data showed consistent improvement over the original data in both the ROM (*p* = 0.014) and ADL (*p* < 0.001) tasks. Overall, ND + Stroke training showed the best performance in both tasks, as well as on the original and augmented data.

Figure S1 in the Supplementary Materials shows the F1-scores for individual participants. This result was obtained from the ND + Stroke training group using the original data. This shows the cross-validated evaluation of each participant in both the ND and Stroke groups. Table 6 summarizes the results with the group mean, standard deviation, and minimum and maximum values for the corresponding participants. Except for a single participant, ND32, who had the lowest F1-score in the ROM task, the ND participants showed consistently higher performance in both the ROM and ADL tasks, and within-subject variability was relatively small. In contrast, stroke patients showed high within-subject variability. In the ROM task, some stroke participants (e.g., Stroke 3, 4, 5, 12, and 13) performed as well as the ND participants, whereas the others performed far below the level. In the ADL task, similar within-subject variability was observed in the stroke group. However, the overall mean performance decreased because the number of movement classes in the ADL task (56 movements) was greater than that of the ROM task (14 movements). This also affected the minimum and maximum values. However, ND participants performed slightly better in the ADL task than they did in the ROM task despite the larger number of classes, as described above. This suggests that the deep learning model has sufficient capacity to extract features from over 61 movement classes if the movements were performed in ordinary patterns. In contrast, the performance drop in the Stroke group suggests that the patients in the group must have had distinct and unusual movement patterns compared to the ND participants.

Additionally, to test a practical use scenario, the results from the ND + Stroke training were evaluated and compared with two sub-datasets: data from all channels (accelerometer + gyroscope) of Sensor 1 (attached to the right wrist; Figure 1, right panel) and data from the accelerometer channels of Sensor 1. We named the first subset S1-all and the second subset S1-acc. For the ND group, compared to the full-sensor data, F1-scores of S1-all decreased by 4.17% and 2.80% for ROM and ADL tasks, respectively, and by 11.6% and 11.3% with S1-acc for ROM and ADL tasks, respectively. However, the performance drop was more salient in the case of the Stroke group. F1-scores of S1-all decreased by 15.9% and 22.5% for ROM and ADL tasks, respectively, and by 25.8% and 33.9% with S1-acc for ROM and ADL tasks, respectively. In summary, the results suggest that a single IMU device attached to a wrist, such as a smartwatch, would suffice for activity recognition in the daily living of non-disabled populations, while stroke patients would benefit from additional body-worn sensors.

**Figure 8:**
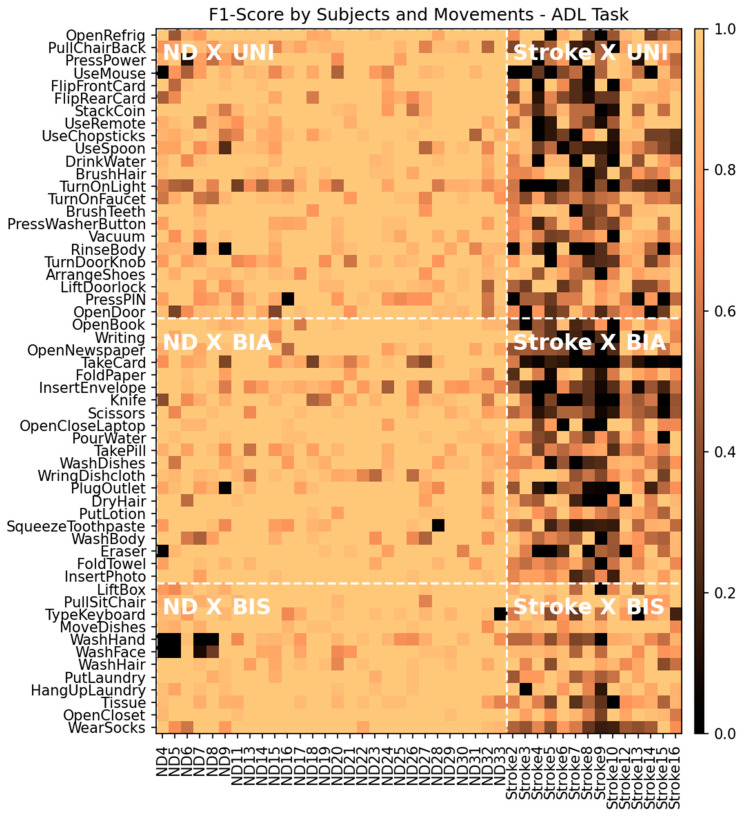
F1-score of individual segments by participants (columns) and movement (rows) for the ADL task. The training group was ND + Stroke with the original data. The areas were divided into six subregions by the combination of the evaluation groups and movement types: ND × UNI, ND × BIA, ND × BIS, Stroke × UNI, Stroke × BIA, and Stroke × BIS.

Figure 8 shows a colormap of the F1-scores of the individual segments in the ADL task with the corresponding participants (columns) and movements (rows). Again, these F1-scores were obtained from the ND + Stroke training group using the original data. The color map is divided into six subregions according to the combination of subject group and movement type to which each segment belongs (denoted by white bold letters and bordered by white dashed lines): ND × UNI, ND × BIA, ND × BIS, Stroke × UNI, Stroke × BIA, and Stroke × BIS. Table 7 summarizes the mean and standard deviation of the F1-scores of the windows in each subregion. The ND group exhibited no significant difference in the F1-scores among the three movement types. In contrast, in the Stroke group, the F1-score of the BIS movement was significantly higher than that of the other two movement types (*p* < 0.001 for all). However, the BIA and UNI for the Stroke group did not differ in F1-score (*p* = 0.226). This indicates that the symmetricity of movement affects the model performance only when movement patterns are abnormal, as in patients with stroke.

**Figure 9:**
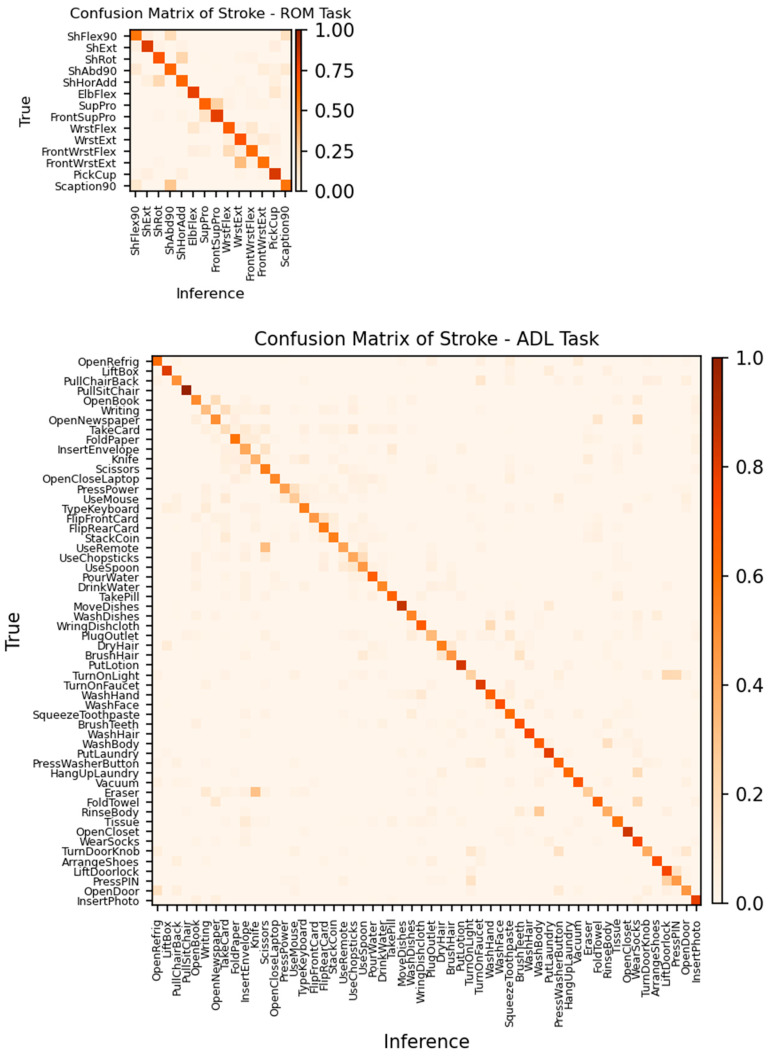
Confusion matrices of patients in the Stroke group for ROM (**upper panel**) and ADL (**lower panel**) tasks. The training group was ND + Stroke with the original data. Movements in the row represent the true class and those in the columns indicate the model inference. A value in a cell is the mean proportion (from 0 to 1) of the row summation.

Figure 9 shows the confusion matrices of the Stroke group for ROM and ADL tasks. The training condition was the same ND + Stroke as that of the original data. In the confusion matrix, the movements in the row represent true classes and those in the column represent the model inference. Therefore, the diagonal elements (row index = column index) are the “right guess”, whereas all off-diagonal elements are considered as the model’s “confusion” or wrong answers. Confusion matrices provide information not only on the global performance metric but also on which movement class was mostly confused with another class by the trained model. Table 8 shows some of the most confused movement pairs for each task.

In the ROM task, the machine learning model encountered difficulties distinguishing between similar movements executed in varying postures. Specifically, in movements involving wrist flexion/extension, discrepancies in posture, such as positioning the elbow bent at 90° adjacent to the torso versus extending the arm fully forward, led to classification inaccuracies even though the movements were simple. In addition, patients with stroke often exhibit abnormal intersegmental synergy. For example, the shoulder and elbow are flexed simultaneously when flexing or abducting the shoulder of the more affected limb. It is difficult for patients to move each segment independently after a stroke. Thus, movements, including shoulder flexion, abduction, and scaption, may be confusing to dissociate. In contrast, in the ADL task, misclassification occurred among movements in which the general actions for the upper and lower arms were similar, except for the distal hand movement. Using a remote controller and using the scissors seemed to be different movements. However, these movements required the upper limbs and forearms to hold the objects while the hand moved in different ways, which was difficult to distinguish using the IMU sensors because they were less sensitive to subtle hand and finger movements.

## 4. Discussion

In the present study, we designed and investigated methods to effectively train and evaluate a deep learning model to classify the movements of stroke patients. Variability in the duration and patterns of stroke patients’ movements leads to challenges in the training and evaluation processes, such as class imbalance and overfitting owing to data sparsity. To address these challenges, we first applied linear interpolation to adjust all segment lengths to a fixed value such that all movement classes had the same number of inputs for training and evaluation. Afterward, in the training process, we tested which training combination of the ND and Stroke groups resulted in the best performance when evaluated in the Stroke group. Contrary to the previous research [7] that a model trained with data including ND does not generalize well to the Stroke group, we found that joint training of ND and Stroke data outperformed the training of Stroke data only for the ADL task. We speculate that the common patterns existing in the data of ND and Stroke patients help reduce overfitting to a smaller volume of stroke data, thus enhancing the generalizability of the trained model. In addition, our findings showed that axis rotation, as a data augmentation method, works effectively and universally across various training and evaluation conditions. It is likely that the axis rotation mimics the variability in hand and arm rotation of individual participants, thereby increasing the experimental data. Finally, we systematically tested how the asymmetry of movements affects evaluation performance in the ND and Stroke groups. The results indicated that for the Stroke group, BIS movements showed better performance than asymmetric movements (BIA and UNI), whereas the ND group did not show any dependency on movement asymmetry. This was mostly because the stroke patients in this study had hemiparesis, and we instructed them to perform asymmetric movements with the affected limb. Therefore, their movement patterns must have been more distinct and diverse, with asymmetric rather than symmetric movements. An investigation of confusion matrices revealed that the most confused pairs of movements included asymmetric movements.

To address the challenges in the classification of stroke movement, we restricted the applicability of the proposed method. To fix the segment lengths, we first manually segment the movements in a continuous data stream. Thus, the model must consider the input as segmented signals, which limits its real-time application when the data stream flows continuously. In future studies, we plan to extend the model to include automatic segmentation functionality. Furthermore, because we used only IMU data in our analysis, our results do not directly indicate how the movement patterns of stroke patients differ in terms of kinematics, such as the joint angle or trajectories of the limbs. The addition of video or marker-based sensor information can complement the current approach. In addition, we tested our methods on a relatively small population of stroke patients with a narrow range of upper-extremity functions. In particular, we did not differentiate between left and right hemiparesis when implementing deep learning. We instructed patients with left hemiparesis to predominantly use their more affected left hand for both BIA and UNI tasks. Some movements, such as writing and using chopsticks or scissors, are highly dependent on the hands. Consequently, using the nondominant and affected left hand could be awkward, resulting in movement patterns that differed from those using the dominant right hand.

Although studies on the application of machine learning models to movement classification in stroke patients exist [7,19,20,47,48,49,50], they included a relatively small set of movements. If the class number of a movement set is small, systematically validating the generalizability of the model to unseen or differently distributed data is difficult. Moreover, a model trained using such small-class data may not be applicable to various daily life activities. We tested our model with two movement sets of different characteristics, ROM and ADL tasks, each containing 14 and 56 upper-extremity movements, respectively, enabling the testing of our methods on diverse scenarios of ADL. A closely related topic is the variability in movement duration. When the number of movement classes is small, the duration tends to be less variable. Thus, the ordinary sliding window technique, which most studies have adopted for deep learning, does not cause serious issues. However, with a larger number of movements, the duration between different movements is more variable; therefore, an imbalanced class distribution is a challenge. We directly addressed this challenge with variable duration with a simple yet efficient linear interpolation to adjust all movement segments to the same duration.

Although prior research has delved into quantifying asymmetric limb usage [27] and examining the accuracy of various ADLs detection through machine learning algorithms in wearable IMU sensors [48], to the best of our knowledge, no study has directly related the asymmetry of movements to model classification performance in stroke populations, which is crucial for hemiparesis rehabilitation. For example, many individuals with stroke tend to use their upper extremities unimanually, particularly relying on the non-paretic side [4]. Correctly detecting UNI, BIA, and BIS activities and providing feedback about these arm usage patterns may encourage increased involvement of the paretic arm and hand in real-life situations. Moreover, the benefits derived from different treatment methods such as constraint-induced movement therapy [1], a task-oriented approach [35], and intensive movement repetition [31] could be sustained after treatments if individuals with stroke continue to use their paretic arm, guided by real-time feedback that results from accurate motion classification.

## 5. Conclusions

The continuous use of the affected arm in daily activities is of significant importance in stroke rehabilitation with hemiparesis. The correct classification of movements from non-intrusive wearable sensor data is the first step for a home rehabilitation system with monitoring and feedback functions. We investigated methods for preprocessing, training, and evaluating a deep learning model to effectively classify the movements of stroke patients. Linear interpolation was used to address variability in movement duration. We found that the joint training of a model with data from the ND and Stroke groups resulted in the best performance compared to training with either group alone. In addition, axis rotation effectively boosts the model performance as a data augmentation technique. Finally, we observed that the model performance in the Stroke group was affected differently by movement asymmetry, suggesting the importance of considering bilaterality and symmetricity when assigning home training movements. Future studies must include automatic segmentation of movements for real-time applications as well as more diverse stroke populations to further investigate the generalizability of the trained deep learning model.

## Figures and Tables

**Table 1:** General characteristics of participants with stroke.

ID	Handedness (R ^a^/L ^b^)	Affected Side (R/L)	Gender (F/M)	Age (Years)	Stroke Onset (Months)	FMA-UE ^c^	MMSE-K ^d^	EHI ^e^
Stroke1	R	R	M	68	19	56	28	90
Stroke2	R	R	F	86	44	58	30	100
Stroke3	R	R	M	73	13	42	27	70
Stroke4	R	R	M	73	14	44	30	100
Stroke5	R	L	M	63	26	59	29	100
Stroke6	R	L	M	69	12	51	30	100
Stroke7	R	L	M	63	44	35	26	70
Stroke8	R	R	M	76	3	33	25	100
Stroke9	R	L	M	59	78	43	28	80
Stroke10	R	L	F	34	13	54	30	100
Stroke11	R	R	F	54	28	63	30	100
Stroke12	R	R	F	61	49	56	30	100
Stroke13	R	R	M	61	29	55	30	100
Stroke14	R	L	M	52	10	66	30	100
Stroke15	R	R	M	65	21	44	30	100

^a^ R: right, ^b^ L: left, ^c^ FMA-UE: Fugl–Meyer Assessment for Upper Extremity, ^d^ MMSE-K: Korean version of Mini-Mental State Examination, ^e^ EHI: Edinburgh Handedness Inventory.

**Table 2:** Example movements in the upper limb motor tasks.

Task	Movement Type	Example Movements
ROM		
	UNI ^a^	Shoulder flexion/extension, external/internal rotation, abduction/adduction
		Elbow and wrist flexion/extension
		Forearm supination/pronation
		Scaption, reaching forward, Ggrasping
ADL		
	UNI	Open the door, turn on the light, brush one’s hair
	BIA ^b^	Put in envelope, fold a towel, open the laptop
	BIS ^c^	Lift the box up, wash one’s face, type on a keyboard

^a^ UNI: unimanual movement, ^b^ BIA: bimanual asymmetric movement, ^c^ BIS: bimanual symmetric movement. All movements in the ADL task belong to a category of “activities of daily living”, drawing upon the examples and definitions cited in the previous research [2,38]. We did not further categorize these ADL movements into “basic” or “instrumental” subsets, as the distinction between these two categories was not a focal point of our study.

**Table 3:** Statistics of segment lengths in time points. Statistics were rounded to integers.

Task	Mean	STD ^a^	Min ^b^	Median	Max ^c^
ROM	1016	492	240	912	3715
ADL	4229	3072	262	3415	25,401

^a^ STD: standard deviation. ^b^ Min: minimum. ^c^ Max: maximum.

**Table 4:** Absolute asymmetry score by movement type.

Task	UNI ^a^	BIA ^b^	BIS ^c^
ROM	4.71 ± 0.80 ^d^	–	–
ADL	2.88 ± 0.92	0.51 ± 0.30	0.22 ± 0.09

^a^ UNI: unimanual, ^b^ BIA: bimanual asymmetric, ^c^ BIS: bimanual symmetric. ^d^ Numbers in a cell represent mean ± standard deviation.

**Table 5:** Evaluated mean F1-scores of the Stroke group trained using three combinations of training groups.

Task		Training Groups		
		ND (Split ^a^)	Stroke (LOSO-CV ^b^)	ND + Stroke (LOSO-CV)
ROM				
	Original data	0.553 ± 0.238 ^c^	0.676 ± 0.105	0.721 ± 0.168
	Augmented data	0.627 ± 0.176	0.709 ± 0.102	0.747 ± 0.126
ADL				
	Original data	0.454 ± 0.162	0.526 ± 0.123	0.603 ± 0.137
	Augmented data	0.512 ± 0.123	0.631 ± 0.096	0.681 ± 0.119

^a^ Split: trained on separate ND data, ^b^ LOSO-CV: trained on leave-one-subject-out cross-validated data. ^c^ Numbers in cells represent means ± standard deviation.

**Table 6:** F1-score by evaluation group in ND + Stroke training with original data.

Task	Evaluation Groups			
	ND		Stroke	
	(mean ± std)	(min/max) ^a^	(mean ± std)	(min/max)
ROM	0.913 ± 0.076	0.575 (ND32)/0.980 (ND8)	0.721 ± 0.168	0.465 (Stroke8)/ 0.939 (Stroke12)
ADL	0.929 ± 0.042	0.829 (ND4)/ 0.980 (ND22)	0.603 ± 0.137	0.359 (Stroke9)/ 0.817 (Stroke12)

^a^ (min/max): the minimum and the maximum F1-scores within each group, along with the corresponding participant IDs.

**Table 7:** F1-score by movement types and evaluation groups from the ADL task.

Movement Types	Evaluation Groups	
	ND	Stroke
UNI	0.918 ± 0.151 ^a^	0.581 ± 0.342
BIA	0.936 ± 0.134	0.541 ± 0.336
BIS	0.938 ± 0.167	0.757 ± 0.266

^a^ Numbers in a cell represent mean ± standard deviation.

**Table 8:** Top 10 confusion pairs.

Task	True ^a^	Inference ^b^	Proportion ^c^
ROM			
	FrontWrstExt (UNI)	WrstExt (UNI)	0.33
	Scaption90 (UNI)	ShAbd90 (UNI)	0.29
	SupPro (UNI)	FrontSupPro (UNI)	0.24
	ShRot (UNI)	ShHorAdd (UNI)	0.22
	ShHorAdd (UNI)	ShRot (UNI)	0.20
	FrontWrstFlex (UNI)	WrstFlex (UNI)	0.19
	ShFlex90 (UNI)	ShAbd90 (UNI)	0.18
	ShFlex90 (UNI)	Scaption90 (UNI)	0.17
	FrontSupPro (UNI)	SupPro (UNI)	0.15
ADL			
	UseRemote (UNI)	Scissors (BIA)	0.32
	Eraser (BIA)	Knife (BIA)	0.31
	RinseBody (UNI)	WashBody (BIA)	0.27
	PressPIN (UNI)	LiftDoorlock (UNI)	0.25
	TurnOnLight (UNI)	LiftDoorlock (UNI)	0.20
	OpenNewspaper (BIA)	WearSocks (BIS)	0.20
	FoldTowel (BIA)	WearSocks (BIS)	0.20
	TurnOnLight (UNI)	PressPIN (UNI)	0.20
	WringDishcloth (BIA)	WashHand (BIS)	0.19

^a^ True: the ground-true movement, ^b^ Inference: the model inference, ^c^ Proportion: the proportion of the confused pair from sum of all inferences.

## Data Availability

The data presented in this study are openly available at https://github.com/youngminoh7/JU-IMU, accessed on 23 November 2023.

## Supplementary Materials

The following supporting information can be downloaded at: https://www.mdpi.com/article/10.3390/s24010210/s1, Table S1: The label names and descriptions for the movements in the ROM task; Table S2: The label names and descriptions for the movements in the ADL task; Figure S1: F1-score of individual participants.
