# Supplementary material for: Investigating Activity Recognition for Hemiparetic Stroke Patients Using Wearable Sensors: A Deep Learning Approach with Data Augmentation
Source: Sensors (Basel). 2023 Dec 29;24(1):210. doi: 10.3390/s24010210 (PMC10781277; doi:10.3390/s24010210)

Table S1. The label names and descriptions for the movements in the ROM task

Type	Label name	Description
**UNI**[b]		
	ShFlex90	Shoulder flexion to 90 degrees[a]
	ShExt	Shoulder extension
	ShRot	Shoulder internal and external rotation
	ShAbd90	Shoulder abduction to 90 degrees
	ShHorAdd	Shoulder horizontal adduction
	ElbFlex	Elbow flexion
	SupPro	Forearm supination and pronation
	FrontSupPro	Forearm supination and pronation with the arm positioned in front of the body
	WrstFlex	Wrist flexion
	WrstExt	Wrist extension
	FrontWrstFlex	Wrist flexion with the arm positioned in front of the body
	FrontWrstExt	Wrist extension with the arm positioned in front of the body
	Pickup	Pick up a cup with a handle
	Scaption90	Scaption to 90 degrees (arm is raised at an angle of approximately 45 degrees forward from the frontal plane of the body)

[a]All items without angle specifications were moved to the participants' maximum range. [b]UNI: unilateral movements

Table S2. The label names and descriptions for the movements in the ADL task

Type	Label name	Description
**BIA**[a]		
	OpenBook	Take the book and open it
	Writing	Writing
	OpenNewspaper	Open a newspaper
	TakeCard	Take the business card and close the cover
	FoldPaper	Fold a paper
	InsertEnvelope	Put in envelop
	Knife	Use a knife
	Scissors	Use scissors
	OpenCloseLaptop	Open and close a laptop cover
	PourWater	Pour water
	TakePill	Open a medicine sachet
	WashDishes	Wash the dishes

	WringDishcloth	Wring out the dishcloth
	PlugOutlet	Plug in an outlet
	DryHair	Dry hair
	PutLotion	Apply lotion after squeezing it out
	SqueezeToothpaste	Squeeze toothpaste
	WashBody	Wash the body with a shower puff
	Eraser	Erase
	FoldTowel	Fold a towel
	ZipUpJacket	Zip up the jacket
	InsertPhoto	Open an album and insert photos
**BIS**[b]		
	LiftBox	Lift the box up and put it down
	PullSitChair	Pull out the chair and sit down
	TypeKeyboard	Type on a keyboard
	MoveDishes	Move dishes
	WashHand	Wash hands
	WashFace	Wash one's face
	WashHair	Wash one's hair
	PutLaundry	Put laundry in and take it out
	HangUpLaundry	Shake out the towel and hang it up
	Tissue	Roll up the tissue
	OpenCloset	Open and close the closet
	WearSocks	Put on socks and take them off
**UNI**[c]		
	OpenRefrig	Open and close refrigerator
	PullChairBack	Pull back the chair
	PressPower	Press power
	UseMouse	Use a mouse
	FlipFrontCard	Flip the business card1
	FlipRearCard	Flip the business card2
	StackCoin	Stack coins
	UseRemote	Use a remote controller
	UseChopsticks	Use chopsticks
	UseSpoon	Use a spoon
	DrinkWater	Drink water
	BrushHair	Brush one’s hair
	TurnOnLight	Turn on the light

TurnOnFaucet	Turn on/off the faucet
BrushTeeth	Brush teeth
OpenWasher	Open washer
PressWasherButton	Press the button on the laundry machine
Vacuum	Vacuum floors
RinseBody	Rinse body
TurnDoorKnob	Turn a knob
ArrangeShoes	Arrange shoes
LiftDoorlock	Lift up/down the door lock
PressPIN	Press the pin number
OpenDoor	Open/close the door

[a]BIA: bilateral asymmetric movements, [b]BIS: bilateral symmetric movements, [c]UNI: unimanual movements

Figure S1. F1-score of individual participants. The training group was ND+Stroke with the original data. The mean score was calculated for all the movements. Error bars represent ± one standard error.

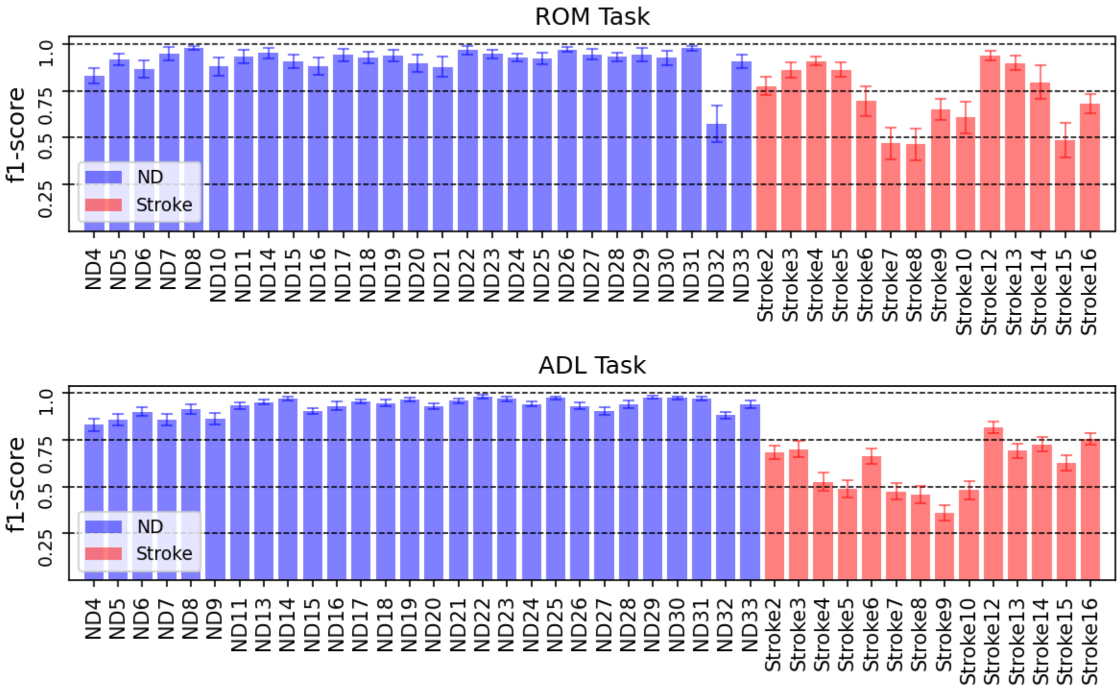

Supplement: Supplementary file 1 [file sensors-24-00210-s001.zip › sensors-2764104-supplementary.pdf]
